# A metabolic signalling role for arginine in liver cancer

**DOI:** 10.1093/lifemeta/load046

**Published:** 2023-11-27

**Authors:** David Sokolov, Lucas B Sullivan

**Affiliations:** Human Biology Division, Fred Hutchinson Cancer Center, Seattle, WA 98109, United States; Human Biology Division, Fred Hutchinson Cancer Center, Seattle, WA 98109, United States

**In addition to their canonical roles in biosynthetic pathways, metabolites can be active participants in oncogenic signalling, but our understanding of these signalling mechanisms is incomplete. In a recent article published in *Cell*, Mossmann *et al.* find a novel signalling role for accumulated arginine in hepatocellular carcinoma (HCC) mediated by the RNA splicing factor and transcriptional modifier RNA-binding protein 39 (RBM39).**

**Figure 1:**
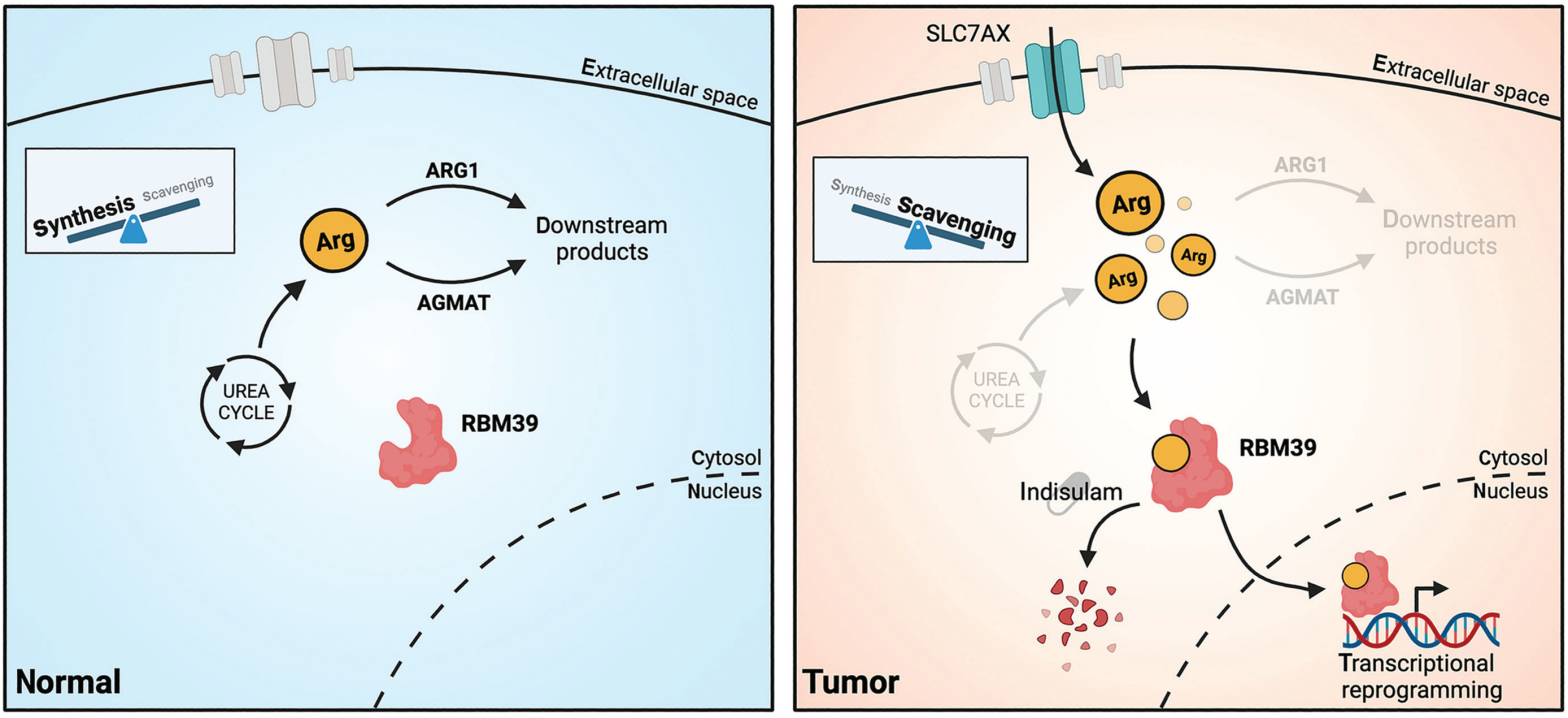
Arginine as an oncogenic signalling molecule in HCC. In non-tumour tissue (left panel), arginine is primarily synthesized *de novo* via the urea cycle, and intracellular arginine levels are kept low by ARG1/AGMAT. Mossmann *et al.* show that in models of HCC (right panel), *de novo* arginine synthesis is downregulated, and cells instead scavenge environmental arginine through SLC family transporters. ARG1/AGMAT levels are also reduced, and accumulated arginine is bound by RBM39, which enacts transcriptional changes promoting tumourigenesis. Created with BioRender.com.

It is now appreciated that cancer cells enact specific metabolic changes to accommodate increased proliferation, create permissive tumour microenvironments, and adapt to physiological stressors, among other functions. When considering the roles of different metabolites in cancer, an informative distinction can be made between so-called *metabolic* roles, in which molecules contribute biomass or energy towards tumour growth, and *signalling* roles, where molecules modify cellular physiology by interfacing with diverse nutrient-sensing pathways. Importantly, a single metabolite can fulfil *both* metabolic and signalling roles; the extent of these roles in different metabolic pathways and their contributions towards tumorigenesis in diverse contexts is fertile ground for new discoveries in cancer metabolism. In a recent publication in *Cell*, Mossmann *et al.* discover and outline a unique signalling role for the amino acid arginine in hepatocellular carcinoma (HCC) via the splicing factor RNA-binding protein 39 (RBM39) [1] (Fig. 1). Their findings—bolstered by a specific RBM39-targeting small molecule that they demonstrate preclinical efficacy in a tumour organoid model—contribute to a growing appreciation of metabolic signalling in cancer and ground the development of new precision therapeutics against RBM39-expressing HCC.

Arginine is a semi-essential amino acid that can be synthesized *de novo* via the urea cycle or scavenged from the environment via several solute carrier (SLC) family amino acid transporters. In addition to its roles in amino acid and urea cycle metabolism, arginine serves a signalling role—one notable example is its binding to CASTOR1 (the cellular arginine sensor for the mechanistic target of rapamycin complex 1 (mTORC1)) to activate the mTORC1 complex and modulate cell growth [2]. Diverse cancer types show deficiencies in the arginine biosynthesis pathway and are auxotrophic for environmental arginine, which has been exploited for mixed clinical benefit since the early 2000s using approaches aimed at enzymatically reducing plasma arginine levels [3, 4]. In particular, HCC, the most common subtype of liver cancer, shows widespread suppression of the urea cycle and corresponding arginine auxotrophy [5]. However, the consequences of these molecular changes on cellular arginine levels and the specific roles of arginine and related metabolites in this context remain incompletely understood.

Mossmann *et al.* began their investigation with a top-down, multi-omics survey of HCC tumour metabolism. Untargeted metabolomics of liver tissues from a genetically engineered mouse model of HCC, driven by liver-specific knockout of tuberous sclerosis complex 1 (TSC1) and phosphatase and tensin homolog (PTEN), revealed broad metabolic alterations in liver tumours compared to controls. Integrating these results with a previously published transcriptomic/proteomic dataset in the same model [6], the authors noted a seemingly paradoxical observation—liver tumours show significantly higher levels of arginine despite transcriptomic downregulation of urea cycle enzymes and reduced levels of the arginine precursors ornithine and citrulline. In agreement with a suppressed urea cycle conferring a dependence on arginine scavenging, they found that several putative arginine transporters of the SLC family were upregulated in tumours. Furthermore, cancerous liver tissues cultured *ex vivo* in radiolabelled arginine showed increased arginine uptake compared to non-cancerous liver samples. These data suggest that arginine metabolism is important for hepatic tumours—but is there a functional requirement for arginine in hepatic tumorigenesis? In an elegant experiment, mice fed arginine-depleted chow were found to form significantly fewer liver tumours than controls, demonstrating that ‘exogenous’ arginine is indeed required for liver tumourigenesis in this model.

The high levels of arginine found in murine liver tumours ought to arise from increased synthesis/acquisition, decreased consumption, or a combination of the two. Having demonstrated apparently impaired arginine synthesis and upregulated arginine import pathways in liver tumours, the authors turned their attention to metabolic pathways that consume arginine and may, therefore, be modulated by tumours to further increase arginine abundance. Chief among these pathways is the synthesis of polyamines (including putrescine, spermidine, and spermine), abundant cationic metabolites that are crucial for cell function but whose specific roles in cancer are not well understood [7]. Two enzymes, arginase 1 (ARG1) and agmatinase (AGMAT), support parallel routes to convert arginine into polyamines, and the authors found in their datasets and on staining that both of these enzymes are downregulated in liver tumours. To functionally link ARG1/AGMAT expression to arginine accumulation, Mossmann *et al*. used adeno-associated viral (AAV) vectors to artificially drive ARG1/AGMAT expression in liver tumours; doing so decreased tumour arginine levels and reduced tumour burden. Interestingly, while polyamine levels were increased in liver tumours, arginine-depleted diets did not reduce their levels, and *ex vivo* tumour tissue demonstrated increased uptake of labelled putrescene in arginine-replete conditions. These facts led the authors to posit that arginine and polyamine pools are decoupled in liver tumours; the additional observation that driving ARG1/AGMAT expression did not affect tumour polyamine levels argues against polyamines representing a significant arginine sink in this context. Thus, while ARG1/AGMAT expression modulates arginine abundance and tumour growth in liver cancer, there appears to be additional metabolic complexity regarding the sources and sinks of arginine to be parsed in future work.

So, liver tumours appear to require high arginine levels, which they achieve by increasing arginine uptake and downregulating the arginine-metabolizing enzymes ARG1 and AGMAT, but what does all this arginine do? To establish a more tractable experimental system, the authors focussed on the SNU-449 human liver cancer cell line, which they demonstrated phenocopies the dependence on low ARG1/AGMAT expression observed in murine hepatic tumours. Drawing on previous results implicating arginine in transcriptional regulation across diverse systems, the authors performed RNA-sequencing on SNU-449 cells with or without ARG1/AGMAT expression. Unexpectedly, this revealed a collection of metabolic genes—spanning diverse pathways including central carbon, amino acid, nucleotide synthesis, and fatty acid metabolism—that were transcriptionally deregulated in ARG1/AGMAT-expressing cells and normalized upon arginine supplementation. Western blotting for several of these targets revealed corresponding changes in protein abundance. Furthermore, the authors verified a subset of these changes in their mouse model of liver cancer. In all, these results suggest that, beyond serving as a precursor for protein synthesis and downstream metabolic pathways, arginine enacts specific transcriptional reprogramming in liver cancer.

By what mechanism does excess arginine exert transcriptional control in liver cancer cell lines and tumours? Towards this point, the authors took inspiration from a previous study in which Geiger *et al*. identified several arginine-sensing transcriptional regulators that mediate pro-survival reprogramming in human T cells [8]. To search for arginine-binding proteins that could function as an intermediary between high arginine levels and transcriptional reprogramming in liver cancer, the team performed a series of immunoprecipitation experiments using arginine-conjugated beads on lysates from SNU-449 cells, mouse liver tumours, and human HCC tumour samples. Strikingly, all three of these pulldowns identified RBM39, an RNA splicing factor known to be upregulated in various cancers but whose particular oncogenic roles remain largely obscure [9]. After several *in vitro* assays confirming that RBM39 specifically binds l-arginine, the authors used mutagenesis to localize the site of arginine binding to the N-terminal 244 amino acids of RBM39.

In their penultimate set of experiments, Mossmann *et al*. sought to determine the functional relevance of an arginine–RBM39 interaction in liver cancer. Focusing on their previously identified arginine-dependent transcriptional signature, they demonstrated that depletion of RBM39 in SNU-449 cells largely normalized the expression of these genes. Moreover, this transcriptional signature was not restored if RBM39 was overexpressed in SNU-449 cells expressing ARG1/AGMAT (which have low arginine levels), and induction of the arginine-responsive gene asparagine synthetase (*Asns*) was abrogated in cells expressing recombinant RBM39 missing its arginine-binding domain. Accordingly, AAV-mediated knockdown of RBM39 in their mouse model reduced liver tumour burden and *Asns* expression, as in SNU-449 cells. In aggregate, these results considerably elevate the arginine–RBM39 interaction from that of a circumstantial observation to a functionally relevant oncogenic event. While the data do not exclude arginine-independent roles of RBM39 in hepatic tumorigenesis, they nonetheless provide a mechanistic link between arginine accumulation and transcriptional remodelling in liver tumours.

It is not often that a novel cancer discovery is met with an available precision therapeutic out of the gates; however, upon outlining a crucial role for arginine-RBM39 in HCC, Mossmann *et al.* were able to leverage one such agent in indisulam. Indisulam is an aryl sulfonamide drug whose anticancer properties had been noted in cell lines and patients since the early 2000s—only recently, though, was it discovered by Han *et al.* that indisulam works by recruiting none other than RBM39 to the cullin 4 (CUL4)-DNA damage-binding protein 1 (DDB1) and CUL4 associated factor 15 (DCAF15) ubiquitin ligase complex and thus targeting it for proteasomal degradation [10]. At several points in their investigation, Mossmann *et al.* used indisulam as a tool compound to interrogate RBM39 in their cell line and mouse models. More impressively, they also demonstrated that indisulam inhibits the growth of patient-derived HCC tumour organoids with higher potency than sorafenib, a kinase inhibitor currently used for the treatment of advance-stage HCC. While more work is warranted to investigate the potential of indisulam as an HCC therapy, these results underscore the translational impact of the authors’ findings. One exciting clinical implication is the prospect of treating arginine-dependent HCC with inhibitors of tumour-specific arginine dependencies as opposed to non-specific arginine depletion, which may have unintended effects on immune cell populations [8].

As with many studies, this work generates at least as many new questions as it answers. Why do HCC tumours suppress the urea cycle, and does the resulting arginine accumulation represent an unintended metabolic phenotype or *bona fide* tumour-driving event? What is the mechanism and functional relevance behind decoupled arginine and polyamine metabolism in hepatic cancer cells? To what extent do the arginine-dependent effects of RBM39 on transcription interact with its arginine-independent RNA splicing functions (which the authors demonstrated are at least partially separable) to contribute to tumourigenesis and the anti-tumour effects of indisulam? Could differences in ARG1/AGMAT expression, or other modifiers of arginine homeostasis, impact responses of HCC patients to RBM39-targeting therapeutics? Future work from this group and others will hopefully clarify the mechanisms involved, as well as determine whether arginine plays a similar signalling role in other urea cycle-deficient cancers. These findings also raise fundamental questions about how fluctuations in intracellular arginine levels might influence the transcriptional and metabolic profiles of non-cancer cells and thereby similarly affect their function in other disease contexts and normal physiology.
